# Stent-Jailing Technique Reduces Aneurysm Recurrence More Than Stent-Jack Technique by Causing Less Mechanical Forces and Angiogenesis and Inhibiting TGF-β/Smad2,3,4 Signaling Pathway in Intracranial Aneurysm Patients

**DOI:** 10.3389/fphys.2018.01862

**Published:** 2019-01-08

**Authors:** Ning Xu, Hao Meng, Tianyi Liu, Yingli Feng, Yuan Qi, Donghuan Zhang, Honglei Wang

**Affiliations:** Department of Neurosurgery, The First Hospital of Jilin University, Changchun, China

**Keywords:** intracranial aneurysm, stent-jailing, stent-jack, angiogenesis, TGF-β, Smad2, 3, 4, endothelial shear stress, microvessel density

## Abstract

**Background:** Stent-jailing and stent-jack are used for stent-assisted coil embolism (SCE) in intracranial aneurysm (IA) therapy, and cause different incidences of IA recurrence. Angiogenesis strongly correlates with aneurysm accumulation. Stent-jack causes higher mechanical forces in cerebral vessels than stent-jailing. Mechanical forces, as well as TGF-β/Smad2,3,4 signaling pathway, may play an important factor in IA recurrence by affecting angiogenesis.

**Methods:** We explored the effects of stent-jailing or stent-jack technique on IA recurrence by investigating mechanical forces, TGF-β/Smad2,3,4 signaling pathway and the incidence of angiogenesis in IA patients. One-hundred-eighty-one IA patients were assigned into stent-jailing (*n* = 93) and stent-jacket groups (*n* = 88). The clinical outcome was evaluated using Glasgow Outcome Score (GOS) and aneurysm occlusion grades. The percentage of CD34^+^EPCs (releasing pro-angiogenic cytokines) in peripheral blood was measured by flow cytometer. Endothelial cells were separated from cerebral aneurysm and malformed arteries via immunomagnetic cell sorting. Angiogenesis was measured by microvessel density (MVD) using anti-CD34 monoclonal antibody staining before using the stent, immediately after surgery and 2 years later. Meanwhile, the mechanical forces in cerebral vessels were determined by measuring endothelial shear stress (ESS) via a computational method. TGF-β and Smad2,3,4 were measured by real-time qPCR and Western Blot. Tube formation analysis was performed to test the relationship between angiogenesis and TGF-β, and the effects of different techniques on angiogenesis.

**Results:** After a 2-year follow-up, 85 and 81 patients from stent-jailing and stent-jack groups, respectively, completed the experiment. Stent-jailing technique improved GOS and reduced aneurysm occlusion grades higher than the stent-jack technique (*P* < 0.05). The counts of CD34^+^EPCs and MVD values in the stent-jailing group were lower than the stent-jack group (*P* < 0.05). ESS values in sent-jailing group were lower than the stent-jack group (*P* < 0.05), and positively correlated with MVD values (*P* < 0.05). TGF-β and Smad2,3,4 levels in sent-jailing group were also lower than the stent-jack group (*P* < 0.05). TGF-β was associated with angiogenesis incidence and stent-jack caused angiogenesis incidence more than stent-jailing.

**Conclusion:** Stent-jailing technique reduces IA recurrence more than stent-jack by causing less mechanical forces, angiogenesis and inhibiting TGF-β/Smad2,3,4 signaling in IA patients.

## Introduction

Intracranial aneurysm (IA) is a common cerebral disease, which involves various organs and becomes more prevalent with high-level morbidity and mortality (Piotin et al., 2018). With the development of medical devices, endovascular stents have been widely used in the prevention of IA (Bakhsheshian et al., 2018; Takahashi et al., 2018). Stent-assisted coil embolization (SCE) techniques are becoming popular and may be feasible and effective for such postoperatively complicated aneurysms (Takeshita et al., 2017).

Stent-jailing technique represents an efficacious adjuvant technique for treating wide-necked persistent trigeminal artery aneurysm (Rong-Bo et al., 2013), in which IAs are “jailed.” For some small aneurysms, stent-jailing technique has been often considered. Nevertheless, the technique should be used carefully and may be unsuccessful occasionally (Yoon et al., 2013). Stent-jack is another technique for complicated aneurysmal treatment. The first coil can be detached into aneurysm dome after the stent is positioned (de Paula Lucas et al., 2008). The technique has been proved to be effective in treating the aneurysms with a ratio of dome height to neck width less than 1.5 (de Paula Lucas et al., 2008).

Complicated IAs are often existed in the internal and middle cerebral artery. Embolization of irregular and complicated IAs is still a challenge. The stent-jailing technique (Tsai et al., 2018) or stent-jack (Lozen et al., 2009) can facilitate efficient embolization of aneurysms. Although stent-jailing and stent-jack techniques are safe and effective for aneurysm therapy but the effects of these techniques on IA recurrence, and associated molecular mechanism remains unknown. Stent-jailing and stent-jack techniques can cause different mechanical forces in cerebral blood vessels. Mechanical forces regulate transforming growth factor-β (TGF-β) and Smads (Maeda et al., 2011). TGF-β is the prototype of structurally related cytokines and control proliferation, differentiation and migration of various cells (Jin et al., 2018). TGF-β has a positive association with the levels of VEGF, and can enhance cellular angiogenesis (Li et al., 2016) while angiogenesis is associated with aneurysm formation (Baumann et al., 2013). SMAD is a family of proteins that play an important role in signal transduction pathways of TGF-β (Huang et al., 2018). Smad2/3 and Smad4 are direct mediators of TGF-β signaling pathway (Ungefroren et al., 2011). Smad2 activates TGF-β type I receptor (TβR-I) and Smad2 phosphorylation is necessary for its nuclear translocation (Rostam et al., 2018). Smad3 is structurally associated with Smad2. Smad4 forms heteromeric complexes with Smad2 or Smad3 and appears to be part of TGF-β signaling pathway (Kretschmer et al., 2003). TGF-β is involved in blood vessel formation while Smad2/3 signaling in endothelial cells is indispensable to keep vessel integrity (Itoh et al., 2012). Angiogenesis is the growth of new blood vessels from the existing vessels and may play an important role in the development of aneurysms (Hoh et al., 2014). IAs can recure or grow after SCE, and TGF-β/Smad2,3,4 signaling pathway, as well as mechanical forces, may play an important factor in the process of angiogenesis.

Stent-Jailing technique and stent-jacket technique may affect the recurrence and regrowth of IA since stent can induce angiogenesis (Zhang et al., 2014), which is associated with aneurysm development (Hoh et al., 2014). Therefore, we explored the effects of stent-Jailing and stent-jacket techniques on the angiogenesis of IA recurrence. Here, we showed that different stent techniques affected angiogenesis of IA patients by affecting mechanical forces in cerebral blood vessels and TGF-β/Smad2,3,4 pathways.

## Materials and Methods

### Reagents

Rabbit anti-Smad2 (ab63576), anti-Smad2 around the phosphorylation site of serine 467 (ab63576), Anti-Smad3 (EP568Y), anti-Smad3 phosphorylated on Serine 423 and 425 (ab52903), anti-Smad4 (EP618Y), anti-CD31 (ab28364), anti-CD34 (ab8536) and goat anti-rabbit HRP (IgG H&L) (ab6721) antibodies were purchased from Abcam trading (Shanghai) co., ltd. (Shanghai, China). Anti-CD 146-coated dynabeads were purchased from Invitrogen Corporation (CA, United States).

### Participants

Before the present experiment, all steps were approved by Human Research Ethics Committee of the First Hospital of Jilin University (Approval No. 2015JLUMPZ), and all patients agreed to sign the informed consent. IAs were measured via digital subtraction angiography (DSA), magnetic resonance angiography (MRA) and computed tomographic angiography (CTA). CTA and MRA can locate the position and show characteristics of IAs. DSA can improve the accuracy of the diagnoses. Severity of IA was evaluated by using Glasgow Outcome Scale (GOS) (Gotoh et al., 1993).

### Inclusion Criteria

The following patients were included: (Piotin et al., 2018) > 18 years; (Takahashi et al., 2018) Modified Rankin Scale (MRS) score < 3; (Bakhsheshian et al., 2018) Clinical symptoms of severe headache, unconsciousness and focal neurological deficits; (Takeshita et al., 2017) the IA identified with CTA.

### Exclusion Criteria

The following excluding criteria were used: (Piotin et al., 2018) the patients had severe medical illness (such as myocardial infarction and psychiatric illness) and or severe sequelae of stroke; (Takahashi et al., 2018) the patients had a history of allergy to aspirin or clopidogrel, and or intracerebral hemorrhage; (Bakhsheshian et al., 2018) besides of aspirin or clopidogrel, the patients took other antiplatelet or anti-inflammatory drugs; and (Takeshita et al., 2017) the patients were pregnant or lactating women.

### Preoperative Treatment

Nimodipine was used and act to inhibit cerebral vasospasm. IA subjects were orally administered with 80-mg clopidogrel (daily) and 100-mg aspirin (daily) 3 days before surgery. Before half-an-hour surgery, patients were injected with one-mg atropine sulfate to prevent gland secretion.

### Patients Grouping

After inclusion and exclusion criteria, 181 IA patients were assigned into stent-jailing (*n* = 93) and stent-jack groups (*n* = 88) based on self-selection after consulting with the brain aneurysm specialists. To eliminate the self-selection bias, a little adjustment was performed to keep no significant difference for IA types and clinical characteristics between two groups after receiving individual agreement (Table 1).

**Table 1:** Clinical characteristics between two groups.

Characteristics			Stent-jailing (*n* = 85)	Stent-jack (*n* = 81)	χ^2^ or *t*-value	*P-*value
Sex, male (%)			50 (58.82)	47 (58.02)	0.011	0.917
Age, years			58.8 ± 22.9	57.6 ± 21.4	0.481	0.258
Hypertension			10 (11.76)	11 (13.58)	0.124	0.725
Diabetes			11 (12.94)	13 (16.05)	0.324	0.569
Dyslipidemia			14 (16.47)	12 (14.81)	0.086	0.769
Coronary artery disease			9 (10.59)	11 (13.58)	0.350	0.554
Smoking			13 (15.29)	15 (18.52)	0.308	0.579
Drinking			12 (14.12)	10 (12.35)	0.113	0.736
Body mass index (kg/m^2^)			17.5-29.3 (23.1 ± 2.7)	19.3-28.6 (21.8 ± 3.4)	0.353	0.060
Cr (mg/dL)			0.42-1.41 (0.80 ± 0.28)	0.46-1.35 (0.83 ± 0.21)	0.026	0.174
CCr (mL/min)			37.6-113.6 (61.4 ± 25.2)	36.3-110.6 (59.3 ± 23.4)	0.554	0.141
Aspirin			73 (85.88)	71 (87.65)	0.113	0.736
Clopidogrel			12 (14.12)	10 (12.35)		
Location	BTA		35 (41.18)	36 (44.44)	0.244	0.970
	VAA		18 (21.18)	17 (20.99)		
	PCAA		19 (22.35)	16 (19.75)		
	PICAA		13 (15.29)	12 (14.81)		
Unruptured/ruptured aneurysm			82/3	79/2	0.003	0.956
GOS score						
1			2 (2.35)	2 (2.47)	0.546	0.969
2			6 (7.06)	7 (8.64)		
3			17 (20)	15 (18.52)		
4			28 (32.94)	26 (32.1)		
5			27 (31.76)	31 (38.27)		
Aneurysm classification						
Fusiform		Simple	4 (4.71)	3 (3.7)	0.685	0.953
		Complex	1 (1.18)	1 (1.23)		
Saccular		No branch	35 (41.18)	34 (41.98)		
		Side Branch	25 (29.41)	23 (28.4)		
		Bifurcation	16 (18.82)	20 (24.69)		
Size		<15 mm	48 (56.47)	45 (55.56)	0.188	0.910
		15-25 mm	24 (28.24)	25 (30.86)		
		>25 mm	13 (15.29)	11 (13.58)		
Stent		Solitaire	63 (74.12)	60 (74.07)	0.001	0.999
		Enterprise	21 (24.71)	20 (24.69)		
		Neuroform	1 (1.18)	1 (1.23)		


*BTA, basilar tip aneurysms; VAA, vertebral artery aneurysm; PCAA, posterior cerebral artery aneurysm; and PICAA, posterior inferior cerebellar artery aneurysm. GOS, Glasgow Outcome Scale. The statistical difference was significant if P < 0.05*.

With CTA, IA location, size and the diameter of parent artery were examined. Based on the results, the angle, path and corresponding stent technique were chosen. The stent-jailing technique was employed in 93 patients. A stent and coils were employed in two microcatheters, and reached IA dome, respectively. The coils were positioned in the IA dome and covered by the stent, and IA was completely packed. Finally, coil microcatheter was withdrawn, and coils were encaged between the stent and parent artery.

Stent-jack technique was also performed in other 88 IA patients according. A first coil in microcatheter was deployed and self-expandable stent was delivered across IA neck, and first coil was detached when the stent was deployed. The technique constrains the coil loops within IA dome before detachment of the first coil.

### Treatment After SCE Intervention

All patients took intraoperative heparinization therapy and orally administrated with 80-mg clopidogrel and 100-mg aspirin daily. Angiography was observed after half year and angiography indicated no symptoms or stent stenosis. The patients would still receive 100-mg aspirin daily during 2-year follow-up.

### Clinical Evaluation of SCE

The embolic and ruptured IA events were evaluated before and immediately postoperative imaging. According to occlusion grades, IAs were classified as complete, sub-complete, partial occlusion and no occlusion grades. Clinical outcomes were measured by using the Glasgow Outcome Scale (GOS) (Tsao et al., 2005). GOS categories 1–3 were regarded as unfavorable and GOS categories 4 and 5 were favorable. The outcome was measured based on IA occlusion grades before and immediately after surgery, and 2 years later.

### Measurement of Peripheral Blood CD34^+^EPCs (Endothelial Progenitor Cells)

CD34^+^EPCs are involved with the release of pro-angiogenic cytokines and associated with pathological angiogenesis (Calzi et al., 2010), and thus the counts of the cells in peripheral blood were measured. One-hundred-microliter peripheral blood was taken from a vein of each patient before and after immediate stent implantation, and 2-year follow-up. Two-microliter mouse anti-human PE-CD34 antibody was added to each tube, and incubated at 4°C overnight; the mixture was centrifuged at 1500 rpm/min at 4°C for 5 min, and the supernatant was discarded. The cells were resuspended in 2 ml PBS, and centrifuged at 2000 rpm/min for 5 min at 4°C, and the supernatant was discarded for 2 times; finally, the blood cells were resuspended in 100 μl of PBS. The cells were stored at 4°C and the number of EPCs was measured on a flow cytometer (Beckman Coulter, Brea, CA, United States).

### Endothelial Cells Isolation

The physicians used real-time X-ray technology to visualize the patient’s vascular system and locate IA inside the blood vessel. A solitaire stent retriever (Covidien, Irvine, CA, United States) was used for biopsy specimens’ retrieval from an occluded IA. Cerebral aneurysm and malformed arteries were isolated from cerebral arties using a cutting plane and separating the surfaces on either side of the plane by using a minimally invasive technique. Endothelial cells were derived from human cerebral aneurysm and malformed arteries, and separated with immunomagnetic cell sorting. Briefly, Anti-CD 146-coated Dynabeads (Invitrogen, CA, United States) were prepared according to manufacturer’s instruction and stored at 4°C. Fifty-mg aneurysms were ground with glass pestle and mortar with one-millilter PBS buffer, 0.1% bovine serum albumin, 0.1% sodium azide, and 0.1% a standard broad-spectrum inhibitor cocktail at 4°C. Ten-microliter FcR-blocking agent (Miltenyi, Bergisch Gladbach, Germany) and 25-microliter antibody-coated Dynabeads were added and mixed thoroughly. The samples were mixed in a mixer for 1 h at 4°C and washed four times with PBS inside the Big Easy Magnet (EasySep, United States) at 4°C. Between each washing procedure, the endothelial cells were flushed out with MACS buffer (PBS with 0.5% BSA and 2 mM EDTA, pH 7.0) 10 times in a 100-μL pipette.

### Microvessel Density (MVD) Assay

Angiogenesis is associated with MVD and can be evaluated by MVD (Zhang et al., 2018). For MVD assay, CD31 is considered to be a marker of MVD (Bösmüller et al., 2018; Xing et al., 2018). Isolated arteriovenous malformation and IA tissues were fixed in 10% formalin and embedded in paraffin blocks. Sections were deparaffinized in xylene and rehydrated in a series of different concentrations of ethanol. After deparaffinization, antigen-retrieval procedure and blocking of endogenous peroxidase, 5-μm sections were incubated for 20 min with CD31 antibody. Subsequently, the sample was incubated with HRP secondary antibody for 10 min, DAB for 15 min and stained with Mayer’s hematoxylin for 5 min. MVD was measured by choosing five regions of each sample at 40× magnification.

### Measurement of Mechanical Forces

The mechanical forces caused by different stent techniques could not be measured *in vivo* exactly. However, the mechanical forces mainly produced shear stress due to blood flow (Ohashi and Sato, 2005), while shear stress could be measured by combining with a computational method. Thus, the mechanical forces in cerebral vessels were analyzed by measuring shear stress. A three-dimension (3D) reconstruction of the target aneurysm vessel was obtained by using an Intravascular Doppler ultrasound catheter (GE Healthcare, Fremont, CA, United States) and biplane cerebral angiogram before and after stent implantation to measure endothelial shear stress (ESS). The catheter was advanced into the cerebral artery and, before starting pull-back, and a biplane cerebral angiogram with contrast injection was performed. The images of the simultaneous electroencephalograph (EEG) signals were recorded. From the data, a computer algorithm was used to calculate the EES via three-dimensional intravascular ultrasound software (Life Imaging Systems, Inc., London, ONT, Canada). The reconstructed cerebral blood vessels were used for 3D geometry calculation and fluid dynamics remodeling. In this way, the values of ESS were measured before and after stent surgery. To avoid different stent length would produce different ESS (LaDisa et al., 2006), six territories proximal to the stent (within 10 mm) were measured.

### Real-Time Quantitative Reverse Transcription-PCR (Real-Time qRT-PCR)

Total RNA was extracted from isolated endothelial cells by using total RNA Isolation Kit (TIANGEN, Beijing, China). The isolated RNA was digested by DNase I (TaKaRa, Dalian, China) according to the manufacturer’s instructions. Two-microliter total RNA was used for reverse transcription to cDNA with the reverse transcription kit (Takara, Dalian, China). The primers for specific genes and β-actin were listed in Table 2. Real-time PCR was performed on LightCycler 2.0 instrument (Roche, Germany). qPCR was performed as follows, 95°C 2 min, and 45 cycles of 95°C for 20 s, 55°C for 15 s, and 72°C for 20 s. Relative gene expression was calculated as 2^-ΔΔCt^.

**Table 2:** The primers used in the present study.

Genes	Primers	Sequences (5′–3′)	Size (bp)
TGF-β	Forward	CAGGGCTTCTCCTACCCCTA	160
	Reverse	GATGGTGGTAGCGTGGGTGG	
Smad2	Forward	CCCCGACACACCGAGATCCT	130
	Reverse	CTGATATATCCAGGAGGTGG	
Smad3	Forward	CCACGCCACACAGAGATCCC	200
	Reverse	AGGTTTGGAGAACCTGCGTC	
Smad4	Forward	ACCATCCAGCATCCACCAAG	200
	Reverse	CCTGGCTGAGGCCCTGATGC	
Actin	Forward	GACATGGAGAAAATCTGGCA	130
	Reverse	AAGGTCTCAAACATGATCTG	


### Western Blot

Frozen samples endothelial cells were lysed in RIPA buffer containing 1% (w/v) of protease/phosphatase inhibitor cocktail (Thermo Scientific, Madison, WT, United States). Protein concentration was measured by BCA detection kit (Beyotime, Beijing, China). The mixed proteins (100 μg) were separated by using SDS-PAGE and transferred to a PVDF membrane for 20 min. Antibodies were incubated overnight at 4°C. The membrane was blocked by using 5% non-fat milk in PBST buffer for 1 h. Secondary antibodies were further incubated and protein bands were visualized chemiluminescence (ECL) system (Bio-Rad, Richmond, CA, United States).

### Tube Formation Analysis

HUVECs were purchased from Shanghai Institutes for Biological Sciences (Cat. No. ECV304) were cultured in RPMI-1640 medium (Gibco Life Technologies, Shanghai, China) supplemented with 10% fetal calf serum at a density of 4 × 10^3^ cells in 96 wells overnight. The cells were treated with 2 μM TGF-β inhibitor (SB505124) (Selleckchem Co., Shanghai, China), or 10 ng/mL TGF-β [Sigma-Aldrich (Shanghai) Trading Co., Ltd., Shanghai, China], the sample supernatants from stent-jailing and stent jack groups for 48 h. A 96-well plate was coated with Matrigel (BD Biosciences) and was cultured at 37°C for half an hour to let Matrigel solidify. HUVECs were plated onto the plate coated by Matrigel. After 20-h culture, the HUVECs were recorded using Olympus IX71 Inverted Compound Microscope (Tokyo, Japan).

### Statistical Analysis

The statistical analysis was carried out by using SPSS 20.0. The possible factors associated with IA risks and postoperative symptoms were analyzed by using univariate and multi-factor analysis. The relationship between the levels of MVD values and ESS values was analyzed using Spearman’s correlation coefficient test.

## Results

### Clinical Characteristics

From April 1st, 2016 to March 1st, 2017, 181 IA patients received SCE therapy, including 97 males and 69 females, aged 35.9–81.7 years. Complete coil embolization was 65 cases (76.47%) and 66 cases (81.48%) in stent-jailing and -jack groups, respectively. After a 2-year follow-up period, 6 and 4 patients withdrew from two groups, respectively. Two and 3 patients were died from two groups because of aneurysmal subarachnoid hemorrhage (SAH), respectively. Thus, 85 and 81 patients completed the study, respectively. The statistical differences for all parameters were insignificant between the two groups (Table 1, *P* > 0.05).

### Primary Outcome

Stent-jailing and stent-jack surgery techniques were employed in IA therapy. Coils successfully covered the aneurysms after SCE surgery and the coils were stable even after a 2-year follow-up period. There were 3 and 5 cases of cerebrovascular vasospasm in stent-jailing group and stent-jack group, respectively. Papaverine (100–115 mg/day) or nimodipine (20–30 mg/day) was administrated to prevent the development of vasospasm. There were 4 and 2 cases of cerebral vascular occlusion in the two groups, respectively. Balloon was deployed to dilate blood vessels. There were 2 cases of embolism coil pop-up in stent-jack group and no case of embolism coil pop-up in stent-jailing group.

### Stent-Jailing Reduced the Incidences of Aneurysm Occlusion Grades More Than Stent-Jack

Aneurysm occlusion grades reflect the severity of ischemic stroke (Qureshi, 2002). The statistical differences for aneurysm occlusion grade were insignificant between two groups before the SCE surgery (Table 3, *P* > 0.05). In contrast, the statistical differences for aneurysm occlusion grade were still insignificant between two groups after immediate SCE surgery (Table 3, *P* > 0.05) but significant after 2-year follow-up (Table 3, *P* < 0.05). The results suggested that stent-jailing was more effective than stent-jack in the prevention of aneurysm occlusion.

**Table 3:** The effects of coil embolism techniques on aneurysm occlusion grades.

Occlusion grades	Stent-Jailing technique	Stent-Jack technique	χ^2^ values	*P*-values
Before aneurysm coil embolization			0.626	0.429
Complete	65 (76.47)	66 (81.48)		
Sub-complete	20 (23.53)	15 (18.52)		
Partially	0 (0)	0 (0)		
No	0 (0)	0 (0)		
After aneurysm coil embolism, cases (%)			0.263	0.967
Complete	2 (2.35)	3 (3.7)		
Sub-complete	12 (14.12)	11 (13.58)		
Partially	18 (21.18)	17 (20.99)		
No	53 (62.35)	50 (61.73)		
2-year follow up			14.865	0.002
Complete	2 (2.35)	8 (9.88)		
Sub-complete	9 (10.59)	21 (25.93)		
Partially	22 (25.88)	23 (28.4)		
No	52 (61.18)	29 (35.8)		


*Complete occlusion, no contrast agents in the aneurysm; sub-complete occlusion, partial contrast agents in aneurysmal dome; partial occlusion, contrast agents filling in the aneurysm dome; and no occlusion, contrast agents is full of in the aneurysm dome.*

### The Effects of Different Stent Techniques on the Counts of CD34^+^EPCs

**FIGURE 1:**
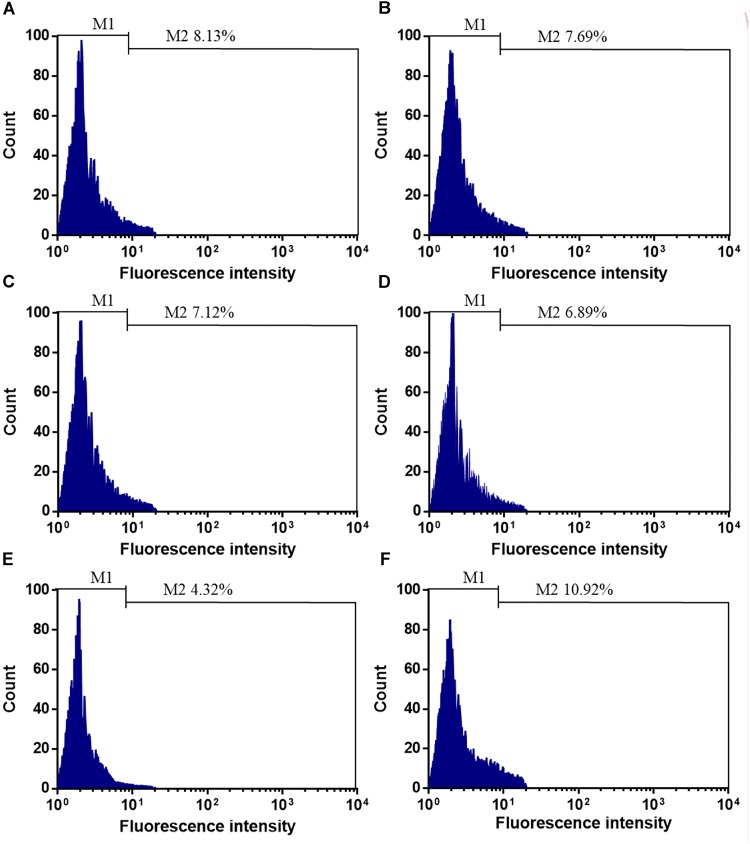
The effects of different stent techniques on the counts of CD34^+^EPCs. **(A)** The effects of stent-jailing techniques on the counts of CD34^+^EPCs before SCE surgery. **(B)** The effects of stent-jack techniques on the counts of CD34^+^EPCs before SCE surgery. **(C)** The effects of stent-jailing techniques on the counts of CD34^+^EPCs after immediate SCE surgery. **(D)** The effects of stent-jack techniques on the counts of CD34^+^EPCs after immediate SCE surgery. **(E)** The effects of stent-jailing techniques on the counts of CD34^+^EPCs after a 2-year follow-up period. **(F)** The effects of stent-jack techniques on the counts of CD34^+^EPCs after a 2-year follow-up period. G, the average percentage of CD34^+^ cells between two groups.

Before SCE surgery and after immediate SCE surgery, the statistical difference for the counts of CD34^+^EPCs was insignificant between stent-jailing and stent-jack groups (Figures 1A–D, *P* > 0.05). After 2-year follow-up, the counts of CD34^+^EPCs in the stent-jailing group were lower than in the stent-jack group (Figures 1E,F, *P* < 0.05). The results suggest that stent-jack increases the counts of CD34^+^EPCs, which may increase the incidence of angiogenesis.

### Stent-Jack Increased More MVD Than Stent-Jailing

**FIGURE 2:**
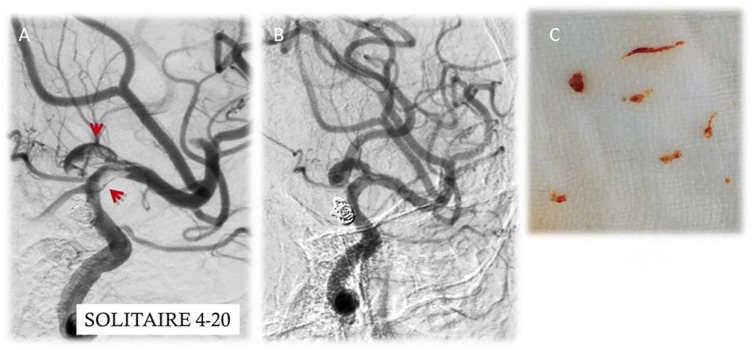
Aneurysm tissues retrieval by a solitaire stent retriever in minimally invasive procedure. **(A)** First-time retrieval of IA biopsy specimens. **(B)** Second-time retrieval of IA biopsy specimens. **(C)** IA biopsy specimens. Red arrow showed the aneurysm.

**FIGURE 3:**
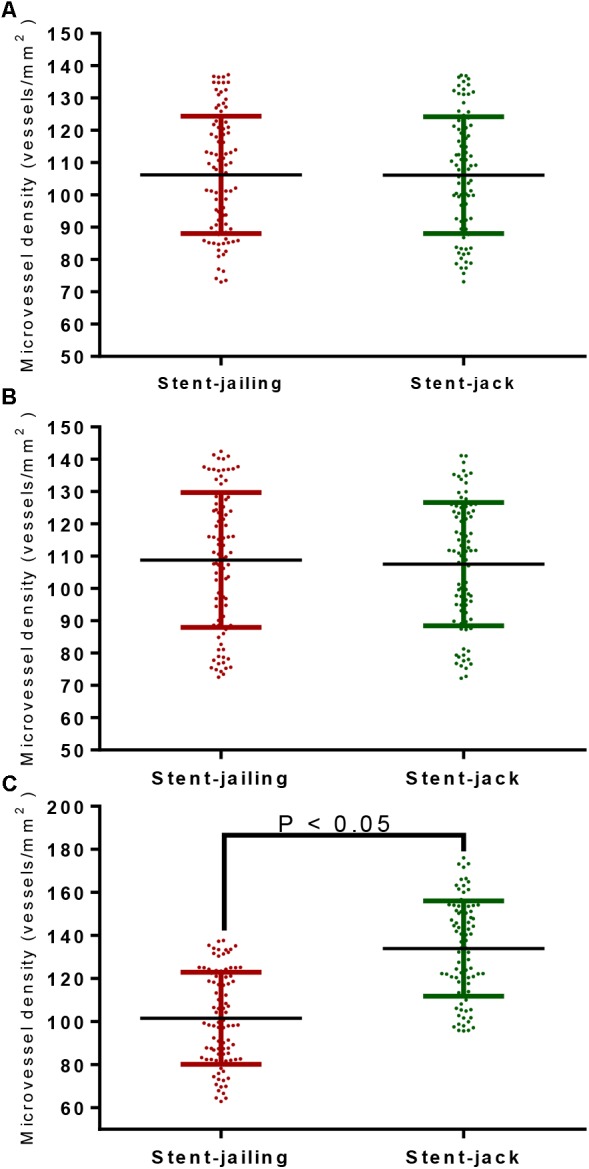
The effects of different stent techniques on microvessel density (MVD) values. **(A)** The effects of different stent techniques on MVD values before SCE surgery. **(B)** The effects of different stent techniques on MVD values after immediate SCE surgery. **(C)** The effects of different stent techniques on MVD values after 2-year follow-up.

After first-time retrieval of IA biopsy specimens (Figure 2A) and second-time retrieval of IA biopsy specimens (Figure 2B), IA biopsy specimens were retrieved well (Figure 2C). Before SCE surgery and after immediate SCE surgery, the statistical difference for MVD levels was insignificant between stent-jailing and stent-jack groups (*P* > 0.05, Figures 3A,B). Mean MVD values were 107.58 ± 35.23 vessels/mm^2^. After 2-year follow-up, MVD values in the stent-jailing group were lower than in the stent-jack group (*P* < 0.05, Figure 3C). Mean MVD values were 100.21 ± 37.45 vessels/mm^2^ in stent-jailing group and 136.72 ± 41.98 vessels/mm^2^ in stent-jack group.

### Stent-Jack Increased ESS Values More Than Stent-Jailing

**FIGURE 4:**
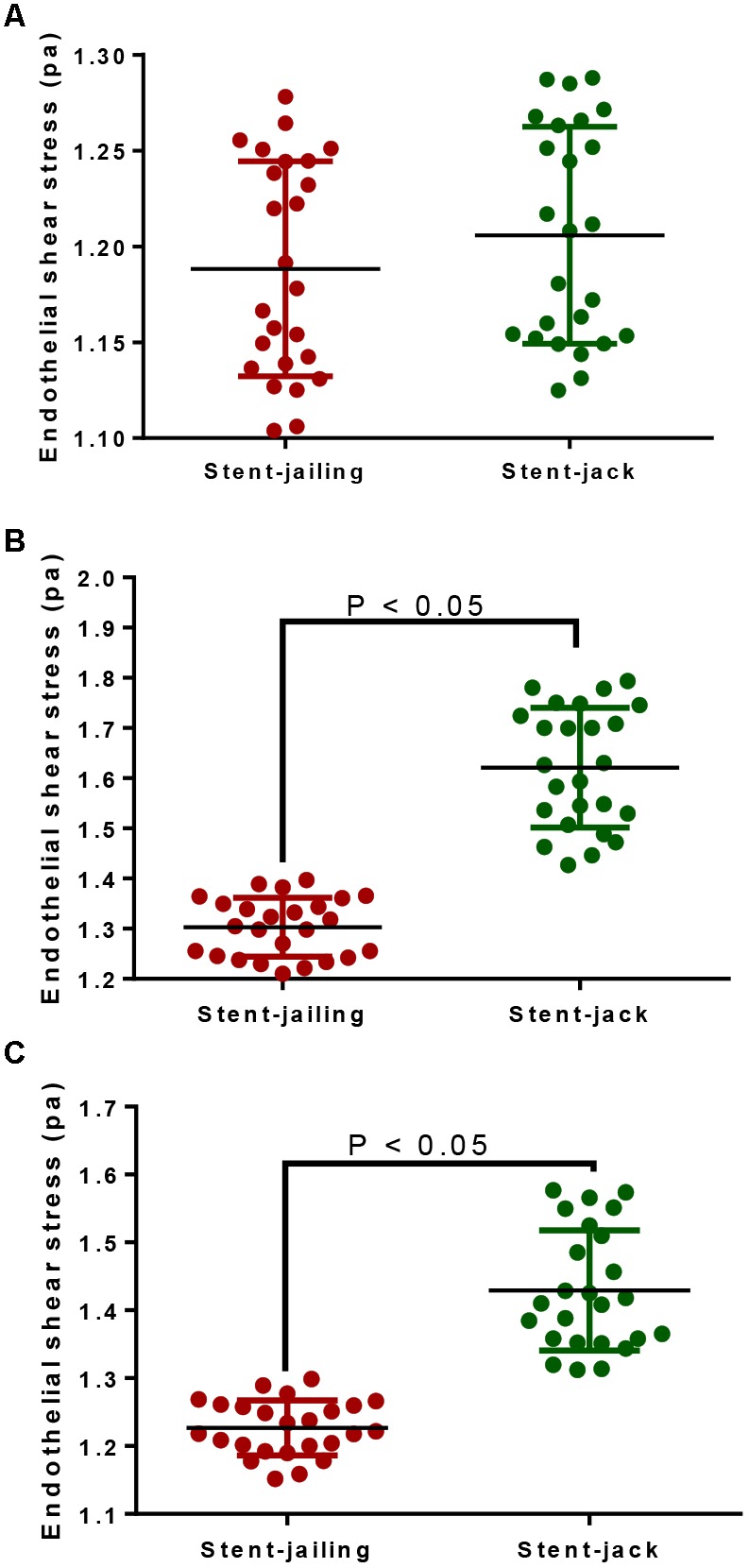
Endothelial shear stress (ESS) values before and after stent implantation within 10-mm segments proximal to the stents. **(A)** ESS values before stent implantation within 10-mm segments proximal to the stents. **(B)** ESS values after immediate stent implantation within 10-mm segments proximal to the stents. **(C)** ESS values after 2-year stent implantation within 10-mm segments proximal to the stents.

In total, 25 IA patients were analyzed from stent-jailing and stent-jack groups, respectively. The statistical difference for ESS was insignificant before stent implantation (*P* > 0.05, Figure 4A). The surgery caused a significant ESS increase in the entire aneurysm lesion after immediate surgery, and the ESS values in stent-jailing group were lower than in stent-jack group (*P* < 0.05, Figure 4B). Similarly, the ESS values in stent-jailing group were still lower than in stent-jack group after 2-year follow-up (*P* < 0.05, Figure 4C).

### MVD Values Had a Positive Association With ESS Values

**FIGURE 5:**
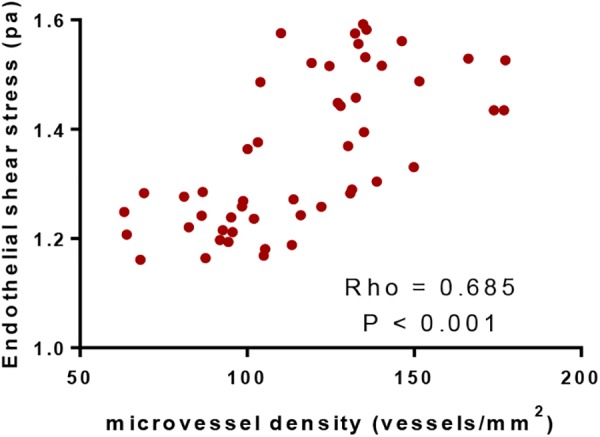
Pearson correlation coefficient analysis of the relationship between the values of microvessel density and endothelial shear stress. There was positive correlation between the two variables if rho values were within 0.5 and 1.

Pearson correlation coefficient analysis showed that ESS values were increased with the increase of MVD values. MVD values had a strong positive association with ESS values since Rho values were more than 0.5 (*P* < 0.05, Figure 5). The results suggested that ESS enhancement increased MVD values, which stand for angiogenic degree (Ames et al., 2016).

### Relative mRNA Levels

**FIGURE 6:**
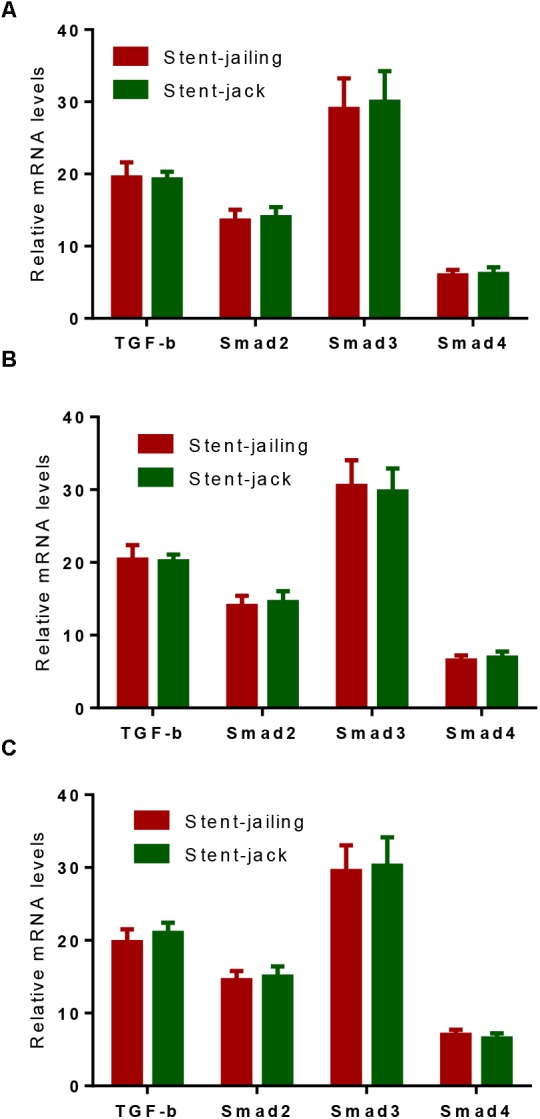
Real-time qPCR analysis of the effects of different stent techniques on relative mRNA levels of TGF-β, Smad2, Smad3 and Smad4. **(A)** The effects of different stent techniques on relative mRNA levels of TGF-β, Smad2, Smad3, and Smad4 before SCE surgery. **(B)** The effects of different stent techniques on relative mRNA levels of TGF-β, Smad2, Smad3, and Smad4 after immediate SCE surgery. **(C)** The effects of different stent techniques on relative mRNA levels of TGF-β, Smad2, Smad3, and Smad4 after a 2-year follow-up period.

Before SCE surgery and after immediate SCE surgery, the statistical difference for relative mRNA levels of TGF-β, Smad2, Smad3, and Smad4 was insignificant between stent-jailing and stent-jack groups (*P* > 0.05, Figures 6A,B). After 2-year follow-up, relative mRNA levels of TGF-β, Smad2, Smad3, and Smad4 were still comparable between stent-jailing and stent-jack groups (*P* > 0.05, Figure 6C). The results suggested that different stent techniques would not change relative mRNA levels of TGF-β, Smad2, Smad3, and Smad4.

### Stent-Jack Promoted TGF-β- Promot Phosphorylation of Smad2 and Smad3

**FIGURE 7:**
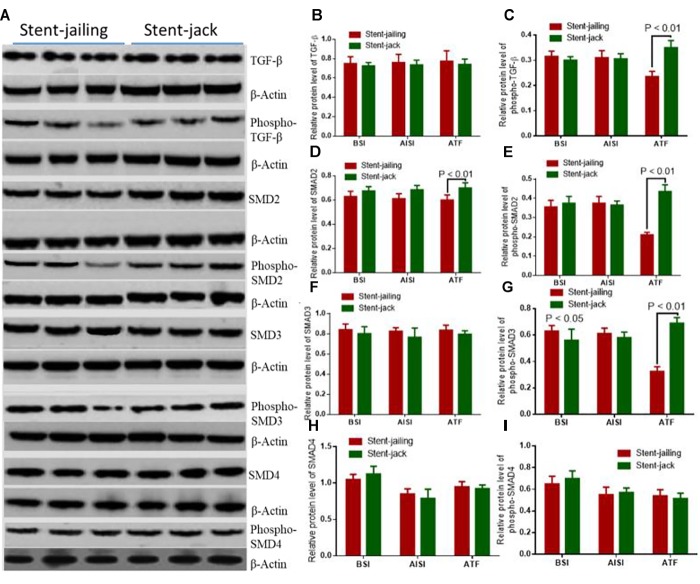
Stent-Jack promoted TGF–β–nt-Jack promoted Smad4 after immediate SCEs. **(A)** The effects of different stent techniques on relative protein levels of TGF-GF-phospho-TGF-β, Smad2, phospho-Smad2, Smad3, phospho-Smad3, and Smad4 before SCE surgery. **(B)** Relative protein levels of TGF-β. **(C)** Relative protein levels of phospho-TGF-β. **(D)** Relative protein levels of Smad2. **(E)** Relative protein levels of phospho-Smad2. **(F)** Relative protein levels of Smad3. **(G)** Relative protein levels of phospho-Smad3. **(H)** Relative protein levels of Smad4. **(I)** Relative protein levels of phosphor-Smad4.BSI, before surgery intervention; AISI, immediately after surgery intervention; ATF, after a 2-year follow-up period. The statistical difference was significant if ^∗^*P* < 0.05 and very significant if ^∗^*P* < 0.01.

Relative protein levels of TGF-β, phospho-TGF-β, Smad2, phospho-Smad2, Smad3, phospho-Smad3 and Smad4 were analyzed by using Western Blot (Figure 7A). Before SCE surgery and after immediate SCE surgery, the statistical difference for relative protein levels of TGF-β (Figure 7B), phospho-TGF-β (Figure 7C), Smad2 (Figure 7D), phospho-Smad2 (Figure 7E), Smad3 (Figure 7F), phospho-Smad3 (Figure 7G), Smad4 (Figure 7H), and Phospho-Smad4 (Figure 7I) were insignificant between stent-jailing and stent-jack groups (*P* > 0.05). After 2-year follow-up, relative protein levels of TGF-β, Smad2, Smad3, and Smad4 were still comparable between stent-jailing and stent-jack groups (*P* > 0.05, Figures 7B,D,F,H,I). However, relative protein levels of phospho-TGF-β (Figure 7C), phospho-Smad2 (Figure 7E) and phospho-Smad3 (Figure 7G) in the stent-jailing group were lower than in the stent-jack group (*P* < 0.05).The results suggested that stent-jack promoted phosphorylated TGF-β-sphoryl phosphorylation of Smad2 and Smad3 when compared with stent-jailing technique.

### Stent-Jailing Potently Reduced More Angiogenesis Than Stent-Jack

**FIGURE 8:**
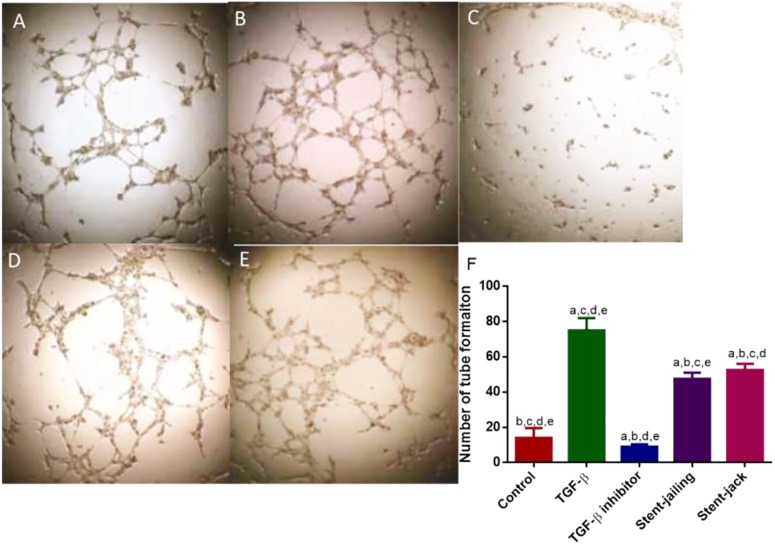
Tube formation of HUVECs among different groups. **(A)** Control group. **(B)** The effect of TGF-β on tube formation in HUVECs. **(C)** The effect of TGF-β inhibitor on tube formation in HUVECs. **(D)** The effect of the supernatants from stent-jailing group on tube formation in HUVECs. **(E)** The effect of the supernatants from stent-jack group on tube formation in HUVECs. **(F)** The average number of tube formation among different groups. ^a^*P* < 0.05 vs. a control group, ^b^*P* < 0.05 vs. a TGF-β group, ^c^*P* < 0.05 vs. a TGF-β inhibitor group, ^d^*P* < 0.05 vs. a stent-jailing group, and ^e^*P* < 0.05 vs. a stent-jack group.

Transforming growth factor-β increased cellular proliferation and promoted angiogenesis when compared with controls (Figures 8A,B,F) (*P* < 0.05). In contrast, SB505124 potently inhibited the proliferation and tube formation of HUVECs (Figures 8C,F) (*P* < 0.05). Comparatively, stent-jailing potently reduced more angiogenesis than stent-jack (Figures 8D–F) (*P* < 0.05). The results proved that stent-jailing and stent-jack may cause different angiogenesis by affecting TGF-β.

## Discussion

Stent-assisted coil embolism is an important strategy in the therapy of IAs. Compared with craniotomy, endovascular embolization has the advantages of less trauma, fewer complications, lower mortality and short procedure time (Cohen et al., 2013). Although endovascular interventional therapy has been shown to be effective, the use of coil embolization alone remains a significant challenge for IA. With the development of techniques, stents have been used to assist coil embolization in the treatment of IAs, particularly wide-neck aneurysms (Feng et al., 2017).

There were 85 aneurysms treated by stent-jailing technology and 81 aneurysm treated by stent-jack in the present work. When stent-assisted embolism is carried out, the choice between the two methods will depend on the expert’s experiences. Stent-jailing method has more advantages than stent-jack. Firstly, catheter placement into aneurysm dome is more difficult after the deployment of stent, especially for closed and small stents. Secondly, like a balloon, the deployed stent will avoid the kickback of the jailing catheter during coil embolism. Thirdly, the occurrence of coil herniation into the parent artery is low. Finally, unintended untangling of coils around the stent-jack can be avoided in stent-jailing technique. The main shortage of stent-jailing technique is that the catheter tip is forced out from aneurysm dome during stent deployment. More coil loops can be deployed to solve the drawback.

Stent-jack can be considered when it is difficult in the application of stent-jailing technique because the stent-jailing technique cannot be adjusted to a satisfactory location. Due to the small opening of aneurysms have been small, scaffold can enter the aneurysm dome via stent-jack. One main drawback for stent-jack is that the detached coil loops may herniate or move, and hang on stent when the stent retrieved.

Peripheral blood CD34^+^ progenitor cells can be stimulated by many cytokines (Brugger et al., 1993). Surgery has been reported to induce the increase in EPC values (Scheubel et al., 2004). We proposed that stent-jack surgery might induce cytokines more than stent-jailing, resulting in high-level of EPCs. CD34^+^EPCs did not increase much after stenting in IA patients between two groups (Figures 1C,D). After 2-year follow-up, the EPC values were increased significantly in stent-jack group (Figure 1F) when compared with other groups, whereas the values was reduced significantly in stent-jailing group (Figure 1E), suggesting other mechanism may be existed for the changes of EPC values. Peripheral blood EPCs can secrete many cytokines associated with angiogenic activities, such as vascular endothelial growth factor (Demetz et al., 2017), hepatocyte growth factor (Iwabayashi et al., 2012) and fibroblast growth factor (Huang et al., 2015).

**FIGURE 9:**
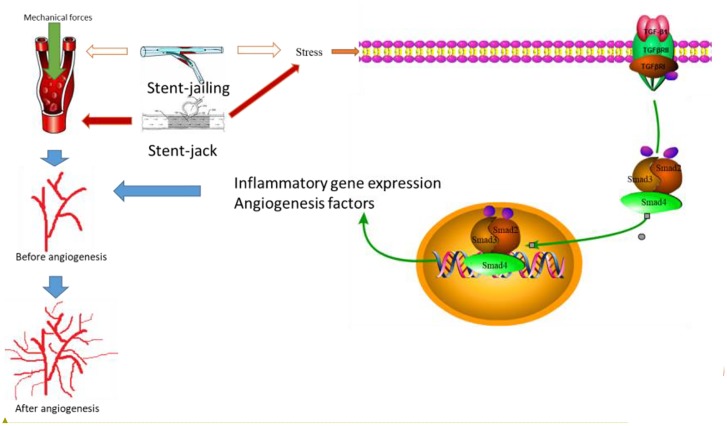
The effects of different stent techniques on angiogenesis. Stent techniques affect angiogenesis via two main ways: different stents will produce different mechanical forces in blood vessel and result in the risk of angiogenesis; different stents will affect TGF-β/Smad2,3,4 signaling pathway differently. Smad2 and Smad3 forms complexes after interacting with TGF–βsignaling pathway diff, which is phosphorylated by T Smad2 and SmaHeteromeric complexes of Smads 2, 3 and 4 can translocated to the nucleus together, resulting in inflammatory gene expression and production of angiogenesis. Red arrows showed an increase in the activity and blank arrows shows no function in the activity.

We showed that stent-jailing rather than stent-jack reduced angiogenesis partly via reducing mechanical forces in cerebral blood vessels or the TGF-β/Smad2,3,4 pathway. The mechanical forces may stimulate endothelial-cell-inducing angiogenesis (Yang et al., 2018). Stent-jack increased ESS values more than stent-jailing technique (*P* < 0.05, Figure 4), suggesting stent-jack caused mechanical forces more than stent-jailing. The results led to the risk of angiogenesis in stent-jack group higher than in stent-jailing group (Figure 9). Phosphorylation of Smad has been widely reported to be associated with the activity of Smad signaling pathway (Seong et al., 2010; Hough et al., 2012). Stent-jailing techniques reduced the phosphorylation of TGF-β, Smad2 and Smad3, and resulted in the reduction of activity of TGF-β/Smad2,3,4 pathway (Figure 9). The decrease in the activity of TGF-β/Smad2,3,4 pathway would inhibit the angiogenesis of the patients (Figure 9) (Pen et al., 2008; Ji et al., 2014; Assis et al., 2015).

There were some limitations of the present work. The interaction of stents with the cerebral endothelial cells in a controlled environment has been widely reported (Sprague et al., 2017; Tefft et al., 2017; Ter Meer et al., 2017). The stent provides the potential attachment and promote the growth of endothelial cells. However, we could not directly detect the associate between the mechanical forces and angiogenesis or the activation of the TGF-β/Smad2,3,4 pathway because the measurement of the mechanical forces applied by each technique is still not feasible *in vivo*. Some long-term complications existed for the patients that undergo aneurysm treatment. First, in-stent thrombosis may be a source of complications because of the angiogenesis caused by mechanical forces. Second, mechanical forces could be technically problematic and induced a further risk of complications. Thus, in-stent thrombosis may be higher. In the present study, aneurysm neck was mainly covered by stents. Although angiogenesis is associated with aneurysm formation (Baumann et al., 2013), angiogenesis may exert good effect to promote endothelialization of the stent to cover the neck of the aneurysm in case of stent-jacket technique and angiogenesis may be required. Cons and pros of angiogenesis were not compared in the present work. Thus, further work is highly required to address these important issues.

## Conclusion

Stent-jailing technique reduced aneurysm occlusion grades more than stent-jack technique after 2-year follow-up by controlling angiogenesis in IA patients. Stent-jailing technique was more effective than stent-jack technique in the treatment of IA patients by reducing mechanical forces and activity of TGF-β/Smad2,3,4 signaling pathway. To confirm the present conclusion, further work in highly demanded in a larger population in the future.

## Author Contributions

NX, HM, and TL performed the experiments, enrolled patients, and analyzed the related data. YF and YQ measured MVD and ESS values. DZ and HW contributed to design the techniques. NX, HM, TL, YF, YQ, and DZ performed the surgery. DZ and HW conceived the experimental plan and wrote the manuscript.

## Conflict of Interest Statement

The authors declare that the research was conducted in the absence of any commercial or financial relationships that could be construed as a potential conflict of interest.
